# Postnatal length and weight growth velocities according to Fenton reference and their associated perinatal factors in healthy late preterm infants during birth to term-corrected age: an observational study

**DOI:** 10.1186/s13052-018-0596-4

**Published:** 2019-01-03

**Authors:** Li Zhang, Yan Li, Shuang Liang, Xiao-Juan Liu, Feng-Ling Kang, Gui-Mei Li

**Affiliations:** 1 Department of Pediatrics, Shandong Provincial Hospital affiliated to Shandong University, 9677, Jingshi Road, Jinan, 250014 Shandong China; 2 Child Health Care Center, Shandong Qianfoshan Hospital affiliated to Shandong University, Jinan, China; 3 Department of Pediatrics, The Second Hospital of Shandong University, Jinan, China; 4 School of Public Health, Shandong University, Jinan, China

**Keywords:** Postnatal, Growth velocity, Catch-up growth, Associated factors, Healthy, Late preterm infants

## Abstract

**Background:**

Optimum early postnatal growth is critical for early and later health of preterm infants. Postnatal length and weight growth velocities and their associated perinatal factors in healthy late preterm infants without restriction of neonatal complications and nutritional problems have not been widely studied.

**Methods:**

As part of ongoing longitudinal follow-up study of growth and development of preterm infants in Shandong Qianfoshan Hospital in China, 599 healthy late preterm infants without neonatal complications and nutritional problems were sampled from 795 preterm infants born in January 2014 to April 2017. Perinatal factors, growth parameters, growth velocities(ΔLengthZ and ΔWeightZ: Z-score changes of length and weight) during birth and term-corrected age were documented. Associated variables of growth velocities were analyzed by bivariate and multivariate regression analyses. Adjusted ΔLengthZ and ΔWeightZ were compared between/among subgroups of associated variables using analysis of covariance. Catch-up growth were defined as ΔLengthZ or ΔWeightZ > 0.67.

**Results:**

The mean ΔLengthZ and ΔWeightZ were 0.28, 0.65, respectively. Catch-up growth of length and weight was ubiquitous(30.7, 46.2%, respectively). Faster length growth velocity was associated with male, larger postmenstrual age(PMA) at birth, younger mother and larger PMA at visit; Faster weight growth velocity was associated with male, unfavorable intrauterine growth status defined by birth weight percentile(Small-for-Gestational-Age(<P10), Appropriate-for-Gestational-Age(P10–90), Large-for-Gestational-Age(>P90)), twin and larger PMA at visit. When adjusted for associated co-variables, weight catch-up growth existed in subgroups of 36 weeks PMA at birth, male, twin and SGA, while AGA almost reached this standard with mean adjusted ΔWeightZ as 0.66. Although none of these subgroups got length catch-up growth standard, infants of 36 weeks PMA at birth had statistically rapider length growth velocity than 34 and 35 weeks PMA at birth subgroups(mean adjusted ΔLengthZs of 34, 35 and 36 weeks subgroups: 0.10, 0.22, 0.38, respectively).

**Conclusions:**

Postnatal length and weight growth velocities of healthy late preterm infants from birth to term-corrected age were much superior than that of Fenton reference, especially for weight, with ubiquitous catch-up growth. Different associated factors for length and weight growth signified the necessity of constructing more detailed growth standards by specific stratification for associated factors.

## Background

Late preterm infants, previously known as Near-Term Newborn Infants, are defined as infants born at 34–0/7 to 36–6/7 weeks of postmenstrual age(PMA) [1]. They consist the largest proportion of preterm infants and 8–9% of total births [2–5]. Nowadays, neonatal services do not routinely follow-up all late preterm infants because to do so would require significant resources [6]. However, late premature birth still interrupts normal in utero fetal development during the last 6 weeks of gestation which is considered as a time-sensitive, irreversible decision point in growth and development [7, 8]. There are accumulating evidences for higher risks of early and later health consequences in late preterm infants [9–13], which would translate into significant medical, emotional, and economic impacts at the population level [14, 15].

Optimum early postnatal growth is critical for improving survival, neurodevelopment and lowering metabolic risks in preterm infants [16, 17]. However, there is no consensus regarding the most suitable growth charts to monitor and evaluate postnatal growth of late preterm infants [18]. Besides, the judgement of optimum postnatal growth is still controversial. Increasing evidences have concluded the inappropriateness to evaluate postnatal growth with intrauterine growth reference(estimates of fetal weight from ultrasonography scans, charts of birth size for PMA) [19–21]. Longitudinal growth values are still of great heterogeneity attributed to conceptual and methodological differences among different studies and thus not necessarily suitable for assessing healthy late preterm infants [18]. Fenton fetal-infant reference has been widely used in evaluating postnatal growth of preterm infants, its computer-assisted graphical smoothing of the disjuncture period around 40 weeks PMA of fetal and infant data sets has been validated by longitudinal growth data of early and moderate preterm infants [19]. However, when evaluated by Fenton reference, the postnatal growth trajectory/velocity and its associated perinatal factors of healthy late preterm infants with adequate control of neonatal comorbidities and nutritional restriction have not been widely studied.

The aim of our study was to monitor the postnatal growth trajectory/velocity according to Fenton reference and to explore the associated factors among healthy late preterm infants from birth to term-corrected age. Comparisons were made among growth parameters of different subgroups stratified by associated variables.

## Methods

### Study design

This study was part of ongoing longitudinal follow-up study of growth and development of preterm infants in Child Health Care Center of Shandong Qianfoshan Hospital in Jinan City, China.

### Subjects

We sampled 599 eligible healthy late preterm infants from 795 preterm infants who were born in January 2014 to April 2017 and got regular health care service in Child Health Care Center. Ethical approval was obtained from the Research and Ethics Committee of Shandong Qianfoshan Hospital before commencement. For all eligible infants, an informed consent was obtained from the parents before enrollment.

**Fig. 1:**
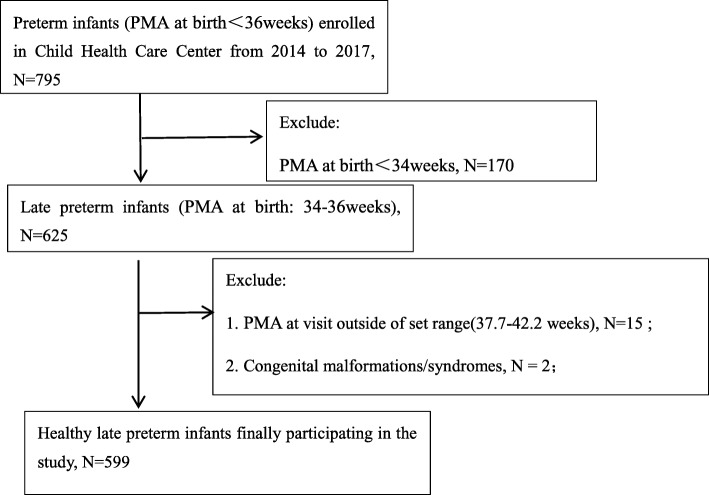
Flow chart showing the recruitment of healthy late preterm infants

The criteria for inclusion and exclusion were as follows: ①PMA at birth: Late preterm birth, defined as 34–36 completed weeks of gestation calculated by last menstrual date, confirmed by early ultrasound measurements; ②No severe neonatal complications: Infants with any neonatal complications(such as severe neonatal asphyxia, hypoxic-ischemic encephalopathy, intracranial hemorrhage, respiratory distress syndrome, necrotizing enterocolitis, etc) which needed parenteral nutrition and intravenous fluid therapy were excluded; ③No congenital malformations and syndromes;④PMA at visit(week): Calculated as PMA at birth(week) + (Date of visit - Date of birth)/7. As the end time-point of this study—PMA at visit should be strictly at 40 weeks(term-corrected age), but it was difficult to control in practice. For better controlling the possible bias, we set the PMA at visit in the range of 37.7–42.3 weeks, equivalent to ±0.5 month chronological age(CA) from the expected date of delivery. Infants with PMA at visit beyond this range were excluded. The flow chart of the recruitment of healthy late preterm infants was shown in Fig. 1.

### Nutrition practice

According to《CSPEN guidelines for nutrition support in neonates》and《Nutrition Practice Care Guidelines for Preterm Infants in the Community 2013》 [22, 23], our preterm infants were fed according to their nutrition risks: Low Nutrition Risk(LNR) was defined as birth weight > 2000 g and no complications; Medium Nutrition Risk(MNR) was defined as birth weight < 2000 g and no complications; High Nutrition Risk(HNR) was defined as birth weight < 2000 g with complications. Before discharge, MNR infants were fed directly with breast feeding plus 3–4 times/day fortified breastfeeding(Breast milk fortifier: Nestle BEBA FM85, German), preterm formulas(Nestle PreNAN, German) was added in case of insufficient breast-milk; LNR infants were fed as full-term born infants: Breastfeeding was encouraged, standard infant formula was added in case of insufficient breast-milk. There were no HNR infants in this study. When discharge(infants got at least 2000 g target weight, with stable feeding and body temperature), parents were encouraged to feed their babies with breast-milk without fortification, standard infant formula was used in case of insufficient breast-milk. Feeding modes after discharge were defined as exclusive breast feeding(EBF), partial breast feeding(PBF) and exclusive formula feeding(EFF) according to current WHO feeding recommendations [24].

### Data collection and growth measurement

Baseline characteristics were obtained retrospectively from birth certificates and parent questionnaires at term-corrected age(at visit): Sex(female 0, male 1), Delivery mode(spontaneous delivery 0, cesarean section 1), Number of fetus(singleton 0, twin 1), Parity(non-primiparity 0, primiparity 1), Nutrition risk(LNR 0, MNR 1), Feeding mode(EBF 0, PBF 1, EFF 2); Intrauterine growth status(Small-for-Gestational-Age(SGA) 0, Appropriate-for-Gestational-Age(AGA) 1, Large-for-Gestational-Age(LGA) 2: defined as birth weight percentile<P10, P10–90, >P90 respectively according to Fenton reference); PMA at birth(week); PMA at visit(week); Education of parents(≤ high school 0, ≥college 1); Age of parents(year).

Growth parameters were measured by experienced nurses within 12 h of birth in obstetrical department and at term-corrected age in Child Health Care Center respectively. Weight was measured with an electronic scales calibrated to 0.05 kg; Length was measured with portable Infantometer(range 30-110 cm, calibrated to 1 mm); Z-scores of growth parameters at birth(Birth WeightZ and LengthZ) and term-corrected age(Term-corrected WeightZ and LengthZ) were calculated by 2013 Fenton-growth-chart calculator [25].

Postnatal growth velocities were defined as changes of Z-scores(ΔLengthZ, ΔWeightZ) from birth to term-corrected age. Growth patterns were defined as catch-down growth(ΔZ < − 0.67), follow-the-curve growth(− 0.67 ≤ ΔZ ≤ 0.67) and catch-up growth(ΔZ>-0.67).

### Statistical analysis

Through bivariate linear regression analysis, we first investigated the association between perinatal factors and ΔLengthZ/ΔWeightZ. After checking for collinearity with a correlation matrix, variables that were marginally significant with a *P* < 0.1 were included in the multivariate linear regression models. Variables were eliminated from the multivariate models using stepwise selection. The final model included only variables with a *P* < 0.05. Comparison were then made between/among subgroups stratified by associated variables using T test, chi-square test, analysis of variance(ANOVA), analysis of covariance(ANCOVA). All statistical analyses were conducted using IBM SPSS Statistics 21 software(Chicago, IL, USA). Two classification variables were presented as number(N) and percentage(%), continuous variables were presented as mean ± standard deviation or mean(95%CI).

## Results

Overall, there are 625 late preterm infants sampled from 795 preterm infants. Twenty six late preterm infants were excluded from this study: ① Overall, there are 625 late15 infants did not get anthropometric measurements during 37.7–42.2 weeks PMA at visit; ② 2 infants had congenital malformations /syndromes (One had congenital achondroplasia, another had severe congenital heart disease); ③ 9 infants suffered by neonatal complications. The remaining 599 healthy late preterm infants were eventually enrolled in this study (Fig. 1). There were no differences in key baseline characteristics among 15 infants with PMA at visit out of set range and those enrolled, but 11 infants who had congenital malformations/syndromes or neonatal complications had obviously smaller birth size than infants enrolled(mean birth LengthZ and WeightZ: − 0.17, − 0.43, respectively, data not demonstrated). The following results were all from 599 enrolled healthy late preterm infants.

1. Baseline characteristics, growth parameters of healthy late preterm infants from birth to term-corrected age.

Baseline characteristics of 599 enrolled subjects were shown in Table 1. There were relatively high proportion of twins(25.0%), the majority were AGA infants(91.2%), primiparity(67.6%), born with caesarean section(65.3%), fed with breast-milk(EBF 60.8%, PBF 28.9%), with high educated parents(> 80% had one of parents received education of college or above), and low proportion of MNR(5.2%).

**Table 1:** Baseline characteristics of 599 healthy late preterm infants^a^

	Total (*N* = 599)
PMA at birth(week)	35.38 ± 0.75
Male, N(%)	337 (56.3)
Twin, N(%)	150 (25.0)
Primiparity, N(%)	405 (67.6)
Caesarean section, N(%)	391 (65.3)
MNR, N(%)	31 (5.2)
Maternal age(year)	30.61 ± 3.95
Paternal age(year)	32.11 ± 4.81
≥College(mother), N(%)	492 (82.1)
≥College(father), N(%)	510 (85.1)
Intrauterine growth status	
SGA, N(%)	30 (5.0)
AGA, N(%)	546 (91.2)
LGA, N(%)	23 (3.8)
Feeding mode	
EBF, N(%)	364 (60.8)
PBF, N(%)	173 (28.9)
EFF, N(%)	62 (10.4)

^a^*Abbreviations:* *PMA* Postmenstrual age, *MNR* Medium nutrition risk, *SGA* Small-for-gestational-age, *AGA* Appropriate-for-gestational-age, *LGA* Large-for-gestational-age, *EBF* Exclusive breast feeding, *PBF* Partial breast feeding, *EFF* Exclusive formula feeding

Growth parameters throughout this study were shown in Table 2. At birth, mean PMA was 35.38 weeks, mean Length and Weight were 47.35 cm, 2.62 kg; LengthZ and WeightZ were 0.26 and − 0.07, respectively. At term-corrected age, the mean PMA(PMA at visit) was 40.84 weeks, mean Length and Weight were 52.92 cm, 4.04 kg; LengthZ and WeightZ were 0.54, 0.58, respectively. Weight and Length growth both demonstrated obviously upward growth compared with intrauterine growth level, of which weight growth velocity was more than twice as fast as length growth(ΔLengthZ: 0.28 ± 0.81, ΔWeightZ: 0.65 ± 0.73).

**Table 2:** Growth parameters of healthy late preterm infants from birth to term-corrected age^a^

	At birth	At term-corrected age	Increment
PMA(week)	35.38 ± 0.75	40.84 ± 0.94	5.46 ± 0.89
Length(cm)	47.35 ± 2.18	52.92 ± 2.25	5.58 ± 2.05
LengthZ	0.26 ± 0.79	0.54 ± 0.91	0.28 ± 0.81
Weight(kg)	2.62 ± 0.42	4.04 ± 0.60	1.41 ± 0.43
WeightZ	− 0.07 ± 0.85	0.58 ± 1.00	0.65 ± 0.73

^a^*Abbreviations:* *PMA* Postmenstrual age, *LengthZ* Z-score of length calculated by Fenton reference, *WeightZ* Z-score of weight calculated by Fenton reference

As for growth patterns(Table 3), the proportion of weight catch-down growth was extremely low(3.0%), only 1/4 of length catch-down growth(13.0%); catch-up growth was ubiquitous in length and weight growth(30.7, 46.2%, respectively), especially for weight, which almost accounted for half of the population.

2. Variables associated with length and weight postnatal growth velocities from birth to term-corrected age.

**Table 3:** Growth patterns of Length and Weight in healthy late preterm infants^a^

	Length	Weight
Catch-down growth, N(%)	78 (13.0)	18 (3.0)
Follow-the-curve growth, N(%)	337 (56.3)	304 (50.8)
Catch-up growth, N(%)	184 (30.7)	277 (46.2)
Sum, N(%)	599 (100.0)	599 (100)

^a^Catch-down growth: Z-score change of growth parameters was lower than − 0.67(ΔZ<-0.67); Follow-the-curve growth: Z-score change was in the range of − 0.67~0.67(− 0.67 ≤ ΔZ ≤ 0.67); Catch-up growth: Z-score change of growth parameters exceeded 0.67(ΔZ > 0.67)

Bivariate linear regression analysis demonstrated the potential associated variables (variables with *P* < 0.1) of ΔLengthZ as Sex(B: 0.177, P: 0.009), PMA at birth(B: 0.188, *P* < 0.001), PMA at visit(B: 0.138, *P* < 0.001), Maternal age(B: -0.016, P: 0.053); the potential associated variable(variables with *P* < 0.1) of ΔWeightZ as Sex(B: 0.218, *P* < 0.001), PMA at birth(B: 0.160, *P* < 0.001), Number of fetus(B: 0.136, P: 0.045), PMA at visit(B: 0.176, *P* < 0.001), Intrauterine growth status(B: -0.274, P: 0.006), Maternal age(B: -0.013, P: 0.071). (Table 4).

**Table 4:** Potential variables associated with postnatal growth velocities by bivariate linear regression analysis^a^

	ΔLengthZ			ΔWeightZ		
Variables	B	Std.Er	*P*-value	B	Std.Er	P-value
Sex(female 0, male 1)	0.177	0.067	0.009	0.218	0.059	0.000
PMA at birth(week)	0.188	0.044	0.000	0.160	0.039	0.000
Number of fetus(singleton 0, twin 1)	0.065	0.077	0.403	0.136	0.068	0.045
Parity (non-primiparity 0, primiparity 1)	0.046	0.071	0.520	−0.028	0.063	0.656
Delivery mode (spontaneous dilivery 0, caesarean section 1)	0.003	0.070	0.970	0.055	0.062	0.375
PMA at visit(week)	0.138	0.035	0.000	0.176	0.031	0.000
Feeding mode(EBF 0, PBF 1, EFF 2)	0.069	0.049	0.165	0.002	0.044	0.961
Intrauterine growth status(SGA 0, AGA 1, LGA 2)	0.040	0.113	0.720	−0.274	0.099	0.006
Nutrition risk(LNR 0, MNR 1)	−0.176	0.151	0.244	0.055	0.133	0.680
Maternal age(year)	−0.016	0.008	0.053	−0.013	0.007	0.071
Paternal age(year)	−0.003	0.007	0.643	−0.002	0.006	0.782
≥ College (mother), N(%)	0.010	0.087	0.912	0.040	0.077	0.602
≥ College (father), N(%)	0.061	0.094	0.515	0.035	0.083	0.669

^a^*Abbreviations:* *PMA* Postmenstrual age, *SGA* Small-for-gestational-age, *AGA* Appropriate-for-gestational-age, *LGA* Large-for-gestational-age, *EBF* Exclusive breast feeding, *PBF* Partial breast feeding, *EFF* Exclusive formula feeding, *LNR* Low nutrition risk, *MNR* Medium nutrition risk, Δ*LengthZ* Z-score change of Length, Δ*WeightZ* Z-score change of Weight

These variables were then further evaluated in a stepwise multivariate regression model using a *P* ≤ 0.05, in order to exclude confounding variables and to explore the actually influential variables on length and weight growth velocities. As demonstrated in Table 5, Sex(B: 0.152, *P*: 0.022), PMA at birth(B: 0.144, P: 0.004), Maternal age(B: -0.017, P: 0.037), and PMA at visit(B: 0.078, P: 0.048), were all associated variables of ΔLengthZ; Sex(B: 0.205, *P* < 0.001), Intrauterine growth status(B: -0.225, P: 0.019), Number of fetus(B: 0.154, P: 0.020), PMA at visit(B: 0.171, *P* < 0.001) were significantly associated with ΔWeightZ, while PMA at birth and Maternal age were excluded(*P* > 0.05).

3. Comparison of growth parameters between/among subgroups of key associated variables (Table 6).

**Table 5:** Variables associated with postnatal growth velocities by stepwise multivariate linear regression analysis^a^

	Variable	B	Std. Err	P-value	95.0% Confidence Interval	
					Lower Limit	Upper Limit
ΔLengthZ	Sex	0.152	0.066	0.022	0.022	0.282
	PMA at birth	0.144	0.049	0.004	0.047	0.240
	Maternal age	−0.017	0.008	0.037	−0.034	− 0.001
	PMA at visit	0.078	0.039	0.048	0.001	0.156
ΔWeightZ	Sex	0.205	0.057	0.000	0.092	0.318
	Intrauterine growth status	−0.225	0.096	0.019	−0.413	− 0.037
	Number of fetus	0.154	0.066	0.020	0.025	0.284
	PMA at visit	0.171	0.030	0.000	0.112	0.230

^a^*Abbreviations:* Δ*LengthZ* Z-score change of Length, Δ*WeightZ* Z-score change of Weight, *PMA* Postmenstrual age

**Table 6:** Comparison of growth parameters between/among subgroups of important associated variables^a^

	PMA at birth			Sex		Number of fetus		Intrauterine growth status		
	34 weeks (*N* = 99)	35 weeks (*N* = 173)	36 weeks (*N* = 327)	Female(*N* = 262)	Male(*N* = 337)	Singleton(*N* = 449)	Twin (*N* = 150)	SGA (*N* = 30)	AGA (*N* = 546)	LGA (*N* = 23)
PMA at birth	–	–	–	35.34 ± 0.77	35.42 ± 0.74	35.39 ± 0.75	35.34 ± 0.75	35.43 ± 0.73	35.37 ± 0.76	35.65 ± 0.65
Male, N(%)	51 (51.5)	95 (54.9)	191 (58.4)	–	–	266 (59.2)	71 (47.3)^κ^	21 (70.0)	304 (55.7)	12 (52.2)
Twin, N(%)	25 (25.3)	49 (28.3)	76 (23.2)	79 (30.2)	71 (21.1)^ε^	–	–	11 (36.7)	139 (25.5)	0 (0)^ƭƞ^
SGA, N(%)	4 (4.0)	9 (5.2)	17 (5.2)	9 (3.4)	21 (6.2)	19 (4.2)	11 (7.3)	–	–	–
AGA, N(%)	93 (93.9)	160 (92.5)	293 (89.6)	242 (92.4)	304 (90.2)	407 (90.6)	139 (92.7)	–	–	–
LGA, N(%)	2 (2.0)	4 (2.3)	17 (5.2)	11 (4.2)	12 (3.6)	23 (5.1)	0 (0)^κ^	–	–	–
Maternal age	29.72 ± 3.26	30.93 ± 4.13^*^	30.72 ± 4.02^*^	30.76 ± 3.95	30.50 ± 3.96	30.62 ± 4.15	30.59 ± 3.29	29.00 ± 2.48	30.59 ± 3.95^ƭ^	33.35 ± 4.21^ƭƞ^
Birth Length(cm)	45.59 ± 2.14	46.93 ± 1.96^*^	48.09 ± 1.94^*§^	47.14 ± 2.13	47.50 ± 2.22^ε^	47.54 ± 2.24	46.75 ± 1.90^κ^	44.55 ± 2.32	47.36 ± 2.00^ƭ^	50.44 ± 1.53^ƭƞ^
Birth Weight(kg)	2.29 ± 0.32	2.51 ± 0.36^*^	2.78 ± 0.40^*§^	2.56 ± 0.41	2.67 ± 0.42^ε^	2.67 ± 0.44	2.47 ± 0.32^κ^	1.95 ± 0.25	2.61 ± 0.33^ƭ^	3.73 ± 0.36^ƭƞ^
Birth LengthZ	0.22 ± 0.84	0.27 ± 0.78	0.26 ± 0.79	0.35 ± 0.75	0.18 ± 0.82^ε^	0.32 ± 0.80	0.05 ± 0.76^κ^	−0.93 ± 0.83	0.27 ± 0.72^ƭ^	1.38 ± 0.58^ƭƞ^
Birth WeightZ	−0.07 ± 0.78	−0.12 ± 0.82	−0.04 ± 0.88	−0.03 ± 0.84	−0.09 ± 0.85	0.03 ± 0.88	− 0.37 ± 0.67^κ^	−1.73 ± 0.44	−0.07 ± 0.63^ƭ^	2.19 ± 0.61^ƭƞ^
PMA at visit(week)	40.00 ± 0.70	40.67 ± 0.73^*^	41.19 ± 0.91^*§^	40.78 ± 0.96	40.89 ± 0.92	40.86 ± 0.94	40.79 ± 0.92	40.85 ± 0.92	40.84 ± 0.94	40.81 ± 0.90
Term-corrected Length(cm)	51.54 ± 1.95	52.60 ± 2.11^*^	53.51 ± 2.19^*§^	52.41 ± 2.17	53.32 ± 2.23^ε^	53.08 ± 2.33	52.46 ± 1.90^κ^	50.41 ± 2.09	52.95 ± 2.13^ƭ^	55.64 ± 1.71^ƭƞ^
Term-corrected Weight (kg)	3.72 ± 0.54	3.94 ± 0.55^*^	4.18 ± 0.59^*§^	3.88 ± 0.58	4.15 ± 0.58^ε^	4.08 ± 0.62	3.89 ± 0.49^κ^	3.29 ± 0.59	4.03 ± 0.54^ƭ^	5.03 ± 0.44^ƭƞ^
Term-corrected LengthZ	0.27 ± 0.91	0.46 ± 0.89	0.67 ± 0.90^*§^	0.54 ± 0.89	0.55 ± 0.92	0.59 ± 0.95	0.39 ± 0.78^κ^	−0.62 ± 0.82	0.55 ± 0.85	1.78 ± 0.68
Term-corrected WeightZ	0.35 ± 1.03	0.47 ± 0.96	0.70 ± 0.99^*§^	0.49 ± 0.96	0.65 ± 1.02	0.64 ± 1.04	0.38 ± 0.82^κ^	−0.98 ± 1.02	0.59 ± 0.86^ƭ^	2.34 ± 0.64^ƭƞ^
ΔLengthZ	0.04 ± 0.90	0.20 ± 0.86	0.41 ± 0.75^*§^	0.19 ± 0.83	0.36 ± 0.80^ε^	0.27 ± 0.82	0.34 ± 0.82	0.30 ± 0.76	0.28 ± 0.82	0.40 ± 0.92
ΔWeightZ	0.42 ± 0.77	0.58 ± 0.74	0.74 ± 0.68^*§^	0.52 ± 0.70	0.74 ± 0.72^ε^	0.61 ± 0.75	0.75 ± 0.61^κ^	0.75 ± 0.84	0.66 ± 0.71	0.15 ± 0.60^ƭƞ^
Adjusted ΔLengthZ^#^	0.10(−0.07, 0.27)	0.22 (0.10, 0.34)	0.38 (0.29, 0.47)^*§^	0.20(0.10, 0.30)	0.35 (0.27, 0.44)^ε^	0.26 (0.19, 0.34)	0.36(0.23, 0.49)	0.23(−0.06, 0.52)	0.28 (0.22, 0.35)	0.45 (0.11, 0.78)
Adjusted ΔWeightZ^#^	0.55 (0.41, 0.70)	0.60 (0.50, 0.71)	0.70 (0.62, 0.78)	0.53 (0.45, 0.62)	0.73 (0.66, 0.81)^ε^	0.61 (0.54, 0.67)	0.76 (0.65, 0.87)^κ^	0.70 (0.45, 0.95)	0.66 (0.60, 0.72)	0.18(−0.11, 0.46)^ƭƞ^

#Adjusted ΔLengthZ and adjusted ΔWeightZ was ΔLengthZ and ΔWeightZ adjusted for respective associated variables by analysis of covariance(for example, ΔLengthZ of different PMA at birth subgroups(34, 35 and 36 weeks PMA at birth) was adjusted for Sex, PMA at visit and Maternal age; ΔWeightZ of different PMA at birth subgroups was adjusted for Sex, PMA at visit, Number of fetus and Intrauterine growth status), other variables were compared by T test, chi-square test, analysis of variance

*:Compared with 34 weeks PMA at birth subgroup, *P* < 0.05; §: compared with 35 week PMA at birth subgroup, *P* < 0.05; ^ε^: compared with female subgroup, *P* < 0.05; κ: compared with singleton subgroup, *P* < 0.05; ƭ: compared with SGA subgroup, *P* < 0.05; ƞ: compared with AGA subgroup, *P* < 0.05

^a^Abbreviations: *PMA* Postmenstrual age, *SGA* Small-for-gestational-age, *AGA* appropriate-for-gestational-age, *LGA* Large-for-gestational-age, *LengthZ* Z-score of Length calculated by Fenton reference, *WeightZ* Z-score of weight calculated by Fenton reference, Δ*LengthZ* Z-score change of length, Δ*WeightZ*, Z-score change of weight

The adjusted ΔLengthZ(adjusted for Sex, Maternal age and PMA at visit) of 34, 35, 36 weeks PMA at birth subgroups were 0.10(− 0.07, 0.27), 0.22(0.10, 0.34), 0.38(0.29, 0.47), respectively, significant difference existed in 34 and 36 weeks, 35 and 36 weeks subgroups(*P* < 0.05), while difference between 34 and 35 weeks subgroups had no statistical significance(*P* > 0.05); adjusted ΔWeightZ(adjusted for Sex, PMA at visit, Intrauterine growth status, Number of fetus) of 34, 35, 36 weeks PMA at birth subgroups were 0.55(0.41, 0.70), 0.60(0.50, 0.71), 0.70(0.62, 0.78), with no statistical significance between any two subgroups (*P* > 0.05).

Male infants had both rapider postnatal growth in length and weight than female infants. The adjusted ΔLengthZ(adjusted for PMA at birth, Maternal age and PMA at visit) of female and male subgroups were 0.20(0.10, 0.30), 0.35(0.27, 0.44), respectively, *P* < 0.05; adjusted ΔWeightZ(adjusted for PMA at visit, Intrauterine growth status, Number of fetus) of female and male subgroups were 0.53(0.45, 0.62), 0.73(0.66, 0.81), respectively, *P* < 0.05.

Twins had significantly rapider weight postnatal growth velocity than singletons, while there was no significant difference in length growth between two subgroups. The adjusted ΔWeightZ(adjusted for Sex, Intrauterine growth status, PMA at visit) of singletons and twins were 0.61(0.54, 0.67), 0.76(0.65, 0.87), *P* < 0.05. The adjusted ΔLengthZ(adjusted for Sex, PMA at birth, Maternal age and PMA at visit) of singletons and twins were 0.26(0.19, 0.34), 0.36(0.23, 0.49), respectively, *P* > 0.05.

There were significant differences in weight postnatal growth velocities in different intrauterine growth status subgroups: SGA and AGA infants had significantly superior weight growth velocities than LGA infants. The adjusted ΔWeightZ(adjusted for Sex, PMA at visit, Number of fetus) of SGA, AGA and LGA subgroups were 0.70(0.45, 0.95), 0.66(0.60, 0.72), 0.18(− 0.11, 0.46), significant difference existed between SGA and LGA (*P* < 0.05), AGA and LGA subgroups(*P* < 0.05), while difference between SGA and AGA subgroups had no statistical significance(*P* > 0.05). Length postnatal growth among three subgroups had no statistical difference. The adjusted ΔLengthZ(adjusted for Sex, PMA at birth, Maternal age and PMA at visit) of SGA, AGA and LGA subgroups were 0.23(− 0.06, 0.52), 0.28(0.22, 0.35), 0.45(0.11, 0.78), *P* > 0.05.

Weight catch-up growth(mean adjusted ΔWeightZ> 0.67) existed in subgroups of 36 weeks PMA at birth, male, twin and SGA infants. AGA infants almost reached this standard with the mean adjusted ΔWeightZ as 0.66. Although length growth of all subgroups demonstrated upward growth without reaching catch-up standard, infants of 36 weeks PMA at birth had statistically rapider length growth velocity than 34 and 35 weeks subgroups.

## Discussion

Early postnatal growth of preterm infants is of great importance since its potential influence for later health. During this critical time-window, the postnatal growth trajectory undoubtedly depends on multiple factors, such as PMA at birth, sex, parental anthropometry, environmental factors (most notably nutrition and disease) and regional, local, ethical and traditional factors, etc., all of which affect postnatal growth through genetic and epigenetic mechanisms, resulting in heterogeneity of growth patterns. Thus, genetics and epigenetics should always be taken into account and considered in the evaluating and monitoring of postnatal life in preterm infants. In our study, the postnatal growth of healthy late preterm infants from birth to term-corrected age could pretty well represent the optimum postnatal growth of local late preterm infants during this critical stage, for the reason that we used similar inclusion criteria as INTERGROWTH-21st Project and WHO Multicentre Growth Reference Study for identifying healthy populations [21, 26]: seemingly free of disease(free of neonatal complications and congenital diseases/syndromes), following current health recommendations(《CSPEN guidelines for nutrition support in neonates》and《Nutrition Practice Care Guidelines for Preterm Infants in the Community 2013》) [22, 23], living in environments unlikely to constrain growth(in an economically developed city of eastern China) and with high-educated parents mastering favorable parenting skills [22, 27]. That is, the postnatal growth of our subjects could represent how healthy late preterm infants in an eastern city of China should grow when there were no detrimental factors(epigenetics) which make the actual postnatal growth deviates from growth potential(genetics). Furthermore, the mean birth weight of our subjects was at the median level of reference fetus according to Fenton reference(Birth WeightZ: − 0.07), which implied the optimum intrauterine nutrition and health status of our subjects.

Previous studies demonstrated obviously less weight and length growth in late preterm infants during first weeks of life than our study. The mean ΔLengthZ and ΔWeightZ of late preterm AGA infants from birth to term-corrected age in Nadia Liotto’s study [28] was − 0.27 and − 0.15, corresponding to our 0.28 and 0.66; The mean ΔLengthZ and ΔWeightZ of late preterm SGA infants in the same study [28] was 0.12 and 0.39, corresponding to our 0.30 and 0.75. The Preterm Postnatal Follow-up Study(PPFS) of the INTERGROWTH-21(st) Project [21] and a latest Chinese preterm cohort [29] both demonstrated slightly less weight increments. The weight increment of 173 late preterm infants in PPFS was 1.20–1.30 kg, slightly less than our 1.42 kg during this period [21]. The weight increment of the Chinese preterm cohort of similar mean PMA at birth(34.9 weeks) was 1.43 kg from birth to 1.40 month CA, corresponding to our 1.42 kg from birth to 1.25 month CA [29]. But this study did not exclude early and moderate preterm infants.

The divergence of inclusion criteria might be the most important contribution to the divergence of postnatal growth. Our subjects were free of neonatal complications and nutrition deficits. In addition, they were sampled from Health Care Center which to some extent excluded those re-hospitalized infants with deviation of growth trajectory. While subjects of most previous studies came from Neonatal Intensive Care Unit(NICU) which inevitably increased the inappropriate proportion of unhealthy infants without approaching growth potential. The similar inclusion criteria and rapid postnatal growth velocities of our study and PPFS could support this assumption [21].

The second possible reason was the differences in feeding and nutrition strategies. In Nadia Liotto’s study, none of the late preterm infants got fortified nutrients, including SGA [28]; while in our study, 5.2%(17 SGA and 14 AGA infants) belonged to MNR and got partially fortified nutrients before discharge (before they got 2000 g target weight), the vast majority(97.4%) of AGA and all LGA infants belonged to LNR and were fed on demand as full-term born infants. Whether this difference in nutrition strategies before discharge contributed to the obvious different growth results was needed for further exploration. However, in this study, there were no significant effects of feeding modes and nutrition risks on postnatal growth during this stage through bivariate linear regression analysis, which might due to the low proportion of MNR and EFF in this study.

Another reason was the differences of the confounding factor—PMA at visit. As was seen in our study, PMA at visit had common obviously positive effects on length and weight growth velocities which implied the prolonged upward growth trends after term-corrected age. It was hard to strictly control PMA at visit at exactly 40 weeks PMA, thus we set the PMA at visit in the range of 37.7–42.3 weeks and used multivariate regression analysis or ANCOVA to control its confounding effects. While previous studies did not specifically depicted PMA at visit of subjects, which might diminish the reliability of comparisons among different studies.

Except for the above-mentioned methodological and nutritional differences between our study and previous studies, regional, local, ethical and traditional factors might also contribute to the divergence of postnatal growth patterns of preterm infants through intricate interactions of genetics and epigenetics. In fact, one of the main areas of dispute in the area of fetal and child growth is whether a single growth reference is representative of growth, regardless of ethnic, region or country origin. Our subjects were all born and living in Jinan City, Shandong Province, which belongs to the economically-developed northern region and has always been the high stature area in China. Therefore, the superior growth trajectory of late preterm infants than Fenton reference and previous studies might to some extent due to the regional and ethnic differences. This assumption should be verified in future studies.

Overall, the rapid postnatal growth velocity was essentially consistent with the superior, close to linear, growth at this stage in Fenton reference and postnatal growth standard of PPFS, which was contrast to the slowing growth velocity of fetus during the weeks before term-corrected age [27]. Although postnatal growth standard of PPFS was constructed through longitudinal data of “healthy” preterm infants born at 27-36 weeks PMA and was designated to be a powerful tool to evaluate postnatal growth of all preterm infants [21], we have noticed the obviously smaller birth size of late preterm infants in PPFS than Fenton reference, newborn size standard[27]and our subjects, of which the gap of growth level continued until term-corrected age. Thus, before a better postnatal growth standard specifically for healthy late preterm infants could be widely used, Fenton reference is still an effective tool with closest growth level and growth velocity compared with other growth references/standards for monitoring postnatal growth of late preterm infants.

A well-known phenomenon associated with postnatal accelerated growth of preterm infants is “catch-up growth”. It is the recovery to the genetic trajectory after a period of growth arrest or delay, pronounced catch­up growth is often seen after severe intrauterine growth restraint(mostly born as SGA) [30]. While the definition of catch-up growth was inconsistency [16, 31]. In our study, it was defined as Z-score changes of growth parameters exceeded the original level by 0.67 according to Fenton reference(ΔZ > 0.67) [16, 30], corresponding with catch-down growth(ΔZ < − 0.67) and following-the-curve growth(− 0.67 ≤ ΔZ ≤ 0.67), because 0.67 SD scores indicate the width between two adjacent percentile curves on standard growth charts(for example, P25 to P50), which could be better applied to all preterm infants and reflect the clinically significant growth fluctuations with possible influences to later metabolism or neurological development [30]. In our healthy late preterm infants, nearly half and 1/3 of infants belonged to weight and length catch-up growth patterns, respectively. In contrast, proportion of catch-down growth was very low, especially for weight(3.0%). Weight catch-up growth was ubiquitous in infants of SGA, AGA, male and those born at 36 weeks PMA. It was contrast to our previously believed notion that the ideal postnatal growth of preterm infants without evidence of intrauterine growth retardation(IUGR) was following the original intrauterine growth curve, that was, ideal extrauterine growth should mimic that in the uterus. It could be inferred that, the concept that optimum postnatal growth velocity of preterm infants should achieve or mimic intrauterine growth velocity was not suitable for healthy late preterm infants. The actual postnatal growth velocities of healthy late preterm infants were much higher than that of fetuses of same PMA, especially for weight. They were even higher than that of Fenton fetal-infant reference, which has obviously higher growth levels and growth velocities than reference fetus of 36-40 weeks PMA [19]. Is this ubiquitous catch-up growth in healthy late preterm infants a physiological phenomenon for better adapting extrauterine environments, or overgrowth which might have potential metabolic risks? Its short and long term implications are still needed for further follow-up studies.

Factors associated with postnatal growth of early and moderate preterm infants have been elucidated in many studies. For example, nutrition accounts for about 50% of the variance in early postnatal growth [32], neonatal complications directly(cause high metabolic state) or indirectly(lead to energy and nutrients deficit) attributed to growth retardation [33]. However, variables associated with optimum postnatal growth of late preterm infants with adequate control of neonatal comorbidities and nutritional restriction have not been widely studied. In healthy late preterm infants without detriments of neonatal complications and nutrition problems, the association of other important perinatal factors(such as PMA at birth, sex) and postnatal growth are much easier to be revealed.

PMA at birth represents the maturity of a preterm infant. The smaller the PMA at birth, the greater risk of mortality, morbidity and growth retardation in early and later postnatal life. However, growth retardation of those born at small PMA is always the consequence of neonatal complications and nutritional problems accompanied by immaturity. Our healthy late preterm infants demonstrated the significantly positive effect of PMA at birth on length growth during birth to term-corrected age, the larger the PMA at birth, the faster the length growth velocity, the most significant difference existed in the 36 weeks subgroup and 34, 35 weeks subgroups. Literatures about the length growth difference in late preterm infants born at different PMA are still scanty. In PPFS, the length growth velocity of 36 weeks subgroup was also obviously superior than that of 34 and 35 weeks subgroups during this period [21]. Inferred from weight postnatal growth, it seems the more impaired intrauterine growth potential(for example, SGA, twin), the faster the postnatal growth. Whether it indicated more potential restriction of intrauterine length growth in late preterm infants born at larger PMA was needed for further exploration. However, at least by Fenton reference, there was no significant difference in Birth LengthZ among different PMA at birth subgroups, thus this assumption was not supported yet. The underlying reasons and implications of length growth difference among different PMA at birth subgroups were needed for further study.

Genetic differences determine different growth potential and trajectories of boys and girls, thus most growth references and standards have set up growth curves for boys and girls respectively. According to Fenton growth curve, male fetus got slightly more weight increment(about 5.8 g/week) and almost identical length growth compared with female during 24-36 weeks PMA in uterus [25]. However, sex differences of postnatal growth do not equate to that of fetal growth even under optimal environments. Our male infants got significantly more weight gain than female infants during birth to term-corrected age and the difference was much more obvious than in uterus(male infants outweighed female by about 23 g/week, while length growth was almost identical). Previous data from early and moderate preterm infants had demonstrated girls’ significantly lower variation of weight gain and incidence of extrauterine growth retardation(EUGR) [4, 34, 35], which were possibly the reflections of girls’ better tolerance of poorer extrauterine environments due to immaturity and subsequently got better growth results [34]. There was no difference in nutrition risks and feeding modes between male and female subgroups, thus nutrition and feeding could not explain the obvious sex differences in postnatal weight growth. Further research is needed to clarify whether it is a physiological phenomenon due to genetic differences or potential higher risk of boys to be overgrowth than girls in healthy late preterm infants.

The incidence of twin pregnancies has increased steadily for the last 40 years due to assisted reproductive technology and increased maternal childbearing age [36]. While there are still few studies on the early postnatal growth of late preterm twin infants. In our study, the weight growth velocity of twins was significantly higher than that of singletons, while length growth velocity was of no significant difference. Even after controlling confounding factors, weight growth differences between twins and singletons were still significant, of which twins could reach the standard of catch-up growth. Whether it implied the relationship of twin pregnancy and potential IUGR is needed for further clarification through maternal-fetal-infant follow-up study.

To our knowledge, the impact of maternal age on the length growth of late preterm infants from birth to term-corrected age has not been reported yet. Although only 1/8–1/9 of the effect of Sex and PMA at birth, the negative effect of maternal age on length growth velocity was still statistically significant. The reasons for the negative effects of maternal age on early postnatal length growth of late preterm infants and its long-term implications are unknown, thus a more detailed study on this effect needs to be carried out by further increasing sample size.

Intrauterine growth status defined as SGA, AGA and LGA according to birth weight percentiles had negative effect on weight growth velocity, significant differences existed in SGA and LGA, AGA and LGA infants. When adjusted for confounding factors, the weight growth velocities of SGA and AGA infants were more than 3.5 times of LGA infants, which exceeded or almost met catch-up growth standards, respectively. There was no significant difference in length growth among three subgroups which might due to the definition of intrauterine growth status(defined by birth weight percentiles rather than birth length percentiles). Postnatal catch-up growth is a widely-known phenomenon in SGA infants which is considered a two edged sword of near and long term health [16, 30, 37]. But the reason for catch-up growth of AGA infants and its implications for later neurological development and metabolic risk are still needed for long-term follow-up study.

It can be seen that, in a certain range, the more potential limitation of intrauterine growth (for example, SGA, twins), the faster the weight postnatal growth velocity under optimum extrauterine environment. Thus a reasonable postulation of this unanimous rapid postnatal growth of healthy late preterm infants was that IUGR might be a ubiquitous phenomenon in late preterm infants, that was, even AGA infants might have a less optimal intrauterine environment which might have caused a reduction in intrauterine weight gain and at the same time preterm birth. Studies estimated that up to 30–50% of preterm labor was associated with IUGR [38, 39]. Once the intrauterine detrimental factors are relieved after birth, there is a natural rebound of postnatal accelerated or even catch-up growth. However, these conclusions mainly came from early and moderate preterm infants, and the proportion of potential IUGR is still hard to define and accurately evaluated.

This study has several implications. It confirmed that optimum early postnatal growth rate of healthy late preterm infants in our center was much rapider than that of Fenton fetal-infant reference. Catch-up growth of weight and length was ubiquitous, especially for weight growth of infants born at 36 weeks PMA, male, twin, SGA and AGA(AGA basically met catch-up growth standard). Under strict control of neonatal complications and implementation of recommended nutrition strategies, perinatal factors such as PMA at birth, sex, intrauterine growth status, maternal age exerted different effects on length and weight growth velocities. The positive effect of confounding factor—PMA at visit on both length and weight growth implies the prolonged catch-up trends after term-corrected age. It implies the inseparable and intricate interactions of genetics and epigenetics on early postnatal growth in late preterm infants. It also signifies the necessity of constructing a more detailed postnatal growth standards stratified by important associated factors such as PMA at birth and sex for better monitoring the optimum postnatal growth of local healthy late preterm infants, and when large population in close region is examined, the use of own local chart should be recommended.

There are some limitations in our study since some other perinatal factors which might be influential factors of postnatal growth were not documented: ①The amount of nutrients and energy intakes which represent the precise effect of nutrition on growth; ② The parental anthropometric data which represents the genetic effects on growth; ③ The indications for preterm delivery categorized as spontaneous, medically indicated or elective delivery to verify intrauterine health conditions except for birth weight percentile [38, 39]; ④The duration of hospitalization which was reflection of postnatal health condition except for neonatal complications [40].

Future studies are needed to improve and enrich above-mentioned factors to observe the most optimal postnatal growth from those most “healthy” late preterm infants. We also need to observe the more detailed growth fluctuations except for the beginning(at birth) and ending(at term-corrected age) during this stage and follow-up for much longer time. Enlargement of healthy SGA and LGA sample sizes is needed to further monitor their specific optimum growth patterns and explore their influential factors. Follow-up studies are also needed for combined effects of intrauterine growth status and early postnatal growth patterns on later metabolic and neurodevelopmental outcomes of healthy late preterm infants for more objective and concrete evidences of developmental origins of health and disease(DOHaD) theory [41].

## Conclusions

In conclusion, healthy late preterm infants demonstrated obviously rapider early postnatal growth velocity than Fenton fetal-infant reference. Catch-up growth was a ubiquitous phenomenon especially for weight growth. Different variables associated with length and weight postnatal growth velocities signified the necessity of construction of more detailed postnatal growth standards stratified by associated variables for better monitoring of postnatal growth. Future follow-up studies are needed for exploring the implication of this rapid postnatal growth and long-term health consequences.

## Abbreviations

AGA: Appropriate-for-gestational-age
ANCOVA: Analysis of covariance
ANOVA: Analysis of variance
CA: Chronological age
EBF: Exclusive breast feeding
EFF: Exclusive formula feeding
EUGR: Extrauterine growth retardation
HNR: High nutrition risk
IUGR: Intrauterine growth retardation
LengthZ: Z-score of length
LGA: Large-for-gestational-age
LNR: Low nutrition risk
MNR: Medium nutrition risk
NICU: Neonatal intensive care unit
PBF: Partial breast feeding
PMA: Postmenstrual age
PPFS: The preterm postnatal follow-up study
SGA: Small-for-gestational-age
WeightZ: Z-score of weight
ΔLengthZ: Z-score changes of length
ΔWeightZ: Z-score changes of weight
